# Reversible Oxygen-Rich Functional Groups Grafted 3D Honeycomb-Like Carbon Anode for Super-Long Potassium Ion Batteries

**DOI:** 10.1007/s40820-022-00892-8

**Published:** 2022-07-21

**Authors:** Na Cheng, Wang Zhou, Jilei Liu, Zhigang Liu, Bingan Lu

**Affiliations:** 1 School of Chemistry and Chemical Engineering, Hunan University, Changsha, 410082 People’s Republic of China; 2 School of Materials Science and Engineering, Hunan University, Changsha, 410082 People’s Republic of China; 3 School of Physics and Electronics, Hunan University, Changsha, 410082 People’s Republic of China

**Keywords:** Honeycomb-like, Super-long, Carbon anode, Potassium-ion batteries

## Abstract

Studies have found that oxygen-rich-containing functional groups in carbon-based materials can be used as active sites for the storage performance of K^+^, but the basic storage mechanism is still unclear. Herein, we construct and optimize 3D honeycomb-like carbon grafted with plentiful COOH/C = O functional groups (OFGC) as anodes for potassium ion batteries. The OFGC electrode with steady structure and rich functional groups can effectively contribute to the capacity enhancement and the formation of stable solid electrolyte interphase (SEI) film, achieving a high reversible capacity of 230 mAh g^−1^ at 3000 mA g^−1^ after 10,000 cycles (almost no capacity decay) and an ultra-long cycle time over 18 months at 100 mA g^−1^. The study results revealed the reversible storage mechanism between K^+^ and COOH/C = O functional groups by forming C-O-K compounds. Meanwhile, the in situ electrochemical impedance spectroscopy proved the highly reversible and rapid de/intercalation kinetics of K^+^ in the OFGC electrode, and the growth process of SEI films. In particular, the full cells assembled by Prussian blue cathode exhibit a high energy density of 113 Wh kg^−1^ after 800 cycles (calculated by the total mass of anode and cathode), and get the light-emitting diodes lamp and ear thermometer running.

**Supplementary Information:**

The online version contains supplementary material available at 10.1007/s40820-022-00892-8.

**Article Highlights**

- A 3D honeycomb-like carbon grafted with plentiful COOH/C = O functional groups (OFGC) is constructed and optimized.
- The study results revealed that the grafting of COOH/C = O functional groups increased specific capacity by forming a C-O-K compound with K, and this reaction is highly reversible.
- The OFGC as anode for potassium-ion batteries achieved a high reversible capacity of 230 mAh g^−1^ after 10,000 cycles (almost no capacity decay) at 3000 mA g^−1^ and an ultra-long stable cycle time over 18 months at 100 mA g^−1^.

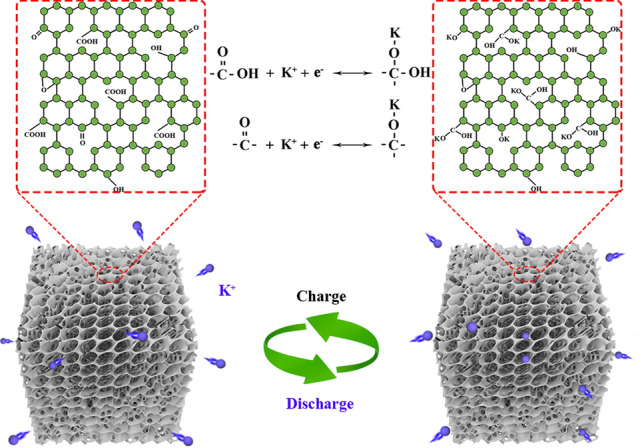

## Introduction

Potassium-ion batteries (PIBs) have been identified as the next generation of large-scale commercial electric energy storage systems due to the cheap potassium salts, lavish potassium resources, and high energy density [1–4]. Nevertheless, the sluggish diffusion kinetics of K^+^ and the degradation of the structure due to the large radius of potassium (0.138 nm) hinder the development of anode electrodes of PIBs [5–9]. Carbon-based materials have acted as an important role in the construction of anode electrode engineering since it was reported that K^+^ can be de/inserted into graphite in 2015 [10–14]. However, the rate performance and cycle stability of graphite as an anode are unsatisfactory due to the limited layer spacing and polarization phenomenon. Moreover, the storage mechanism of K^+^ in graphite anode is mainly de/intercalation behavior, and its capacity is greatly limited (theoretical capacity is 279 mAh g^−1^ when formed KC_8_ compound) [15–18]. Recently, in-depth exploration of the introduction of rich heteroatomic doping (S, N, O, etc.) has been proved to be an effective scheme to improve the electrochemical performance of carbon-based materials by introducing defects and active sites, widening the layer spacing, increasing the ion adsorption capacity, and shortening the ion diffusion path [19–22].

**Scheme 1:**
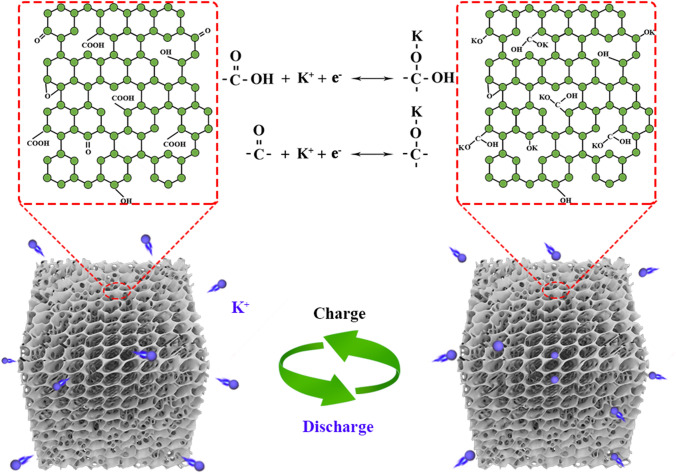
Schematic illustration of reversible potassium storage reaction mechanism of oxygen-rich functional groups (COOH/C = O) in 3D honeycomb-like OFGC hybrid

Among the numerous heteroatom doping, the driving mechanism of oxygen doping is to introduce various oxygen-containing functional groups and structural defects into the carbon framework, which can be used as active sites for K^+^ storage [23, 24]. Therefore, the introduction of suitable oxygen-rich functional groups into carbon-based materials to enhance the redox activity required by electrochemical reactions is the key to obtaining more K^+^ storage. Notably, the grafting of rich oxygen functional groups may increase the impedance of carbon electrodes, but can significantly improve the energy storage capacity of the carbon-based electrode, indicating that the reduction in electrode conductivity does not affect the energy storage efficiency of oxygen functional groups [25]. In addition, oxygen functional groups can also regulate the interface composition of the electrolyte by changing the physical and chemical properties of an electrode material such as defect, edge, structure, and electrical conductivity [26–28]. It has been reported that C = O and COOH functional groups can contribute to the formation of the highly conductive, complete, and robust inorganic SEI films [25]. Nevertheless, the storage mechanism behind the increase in capacity of carbon-based materials grafted by different oxygen functional groups remains unclear. Therefore, it is indispensable to explore the potassium-storage mechanism of oxygen-rich functional groups for promoting the development of carbonaceous materials electrodes in PIBs (Scheme 1).

Herein, inspired by the natural organisms, a 3D honeycomb-like carbon material with plentiful COOH/C = O functional groups (OFGC) was constructed and used as an anode for PIBs. The stable 3D honeycomb-like structure with continuous and interconnected large pore structure is an ideal scaffold for relieving volume expansion caused by multiple (de) potassiation processes. And the coarsely connected nanosheets with a mesoporous structure accelerate the infiltration of electrolyte and K^+^ transport. Moreover, the grafting of the COOH/C = O functional groups improves the reversible specific capacity of the electrode by forming a C-O-K compound with potassium, participating in the regulation of SEI composition, and forming robust SEI films. Thus, the OFGC-600 electrode exhibited extraordinary electrochemical properties, a high initial charge capacity of 454 mAh g^−1^ at 50 mA g^−1^, an ultra-long operating time over 18 months, and maintaining a reversible capacity of approximately 360 mAh g^−1^ at 100 mA g^−1^, and lasting cyclability at 3000 mA g^−1^ (almost no capacity decay after 10,000 cycles). Particularly, the full cells assembled by Prussian blue (PB) cathode obtained a higher energy density of 113 Wh kg^−1^ after 800 cycles, and get the electronic equipment (light-emitting diodes (LED) light, ear thermometer, etc.) running. This work provides new insights for the early commercial application of novel carbon-based materials in PIBs.

## Experimental Section

### Reagents

The trisodium citrate dihydrate (99.9%), hydrochloric acid (37%), K_4_Fe(CN)_6_, FeCl_3_, and potassium metal (99.9%) were purchased from Sigma-Aldrich. Ketjen black, CMC, PVDF, Cu foils, Al foils, potassium bis(fluorosulfonyl)imide (KFSI), Super P, and DME were supplied by Shanghai Songjing Energy Technology Limited. All chemicals are not treated before being directly used.

### Synthesis of Oxygen-rich Functional Groups Carbon and Prussian blue Products

The synthesis method of the OFGC materials was facilely prepared by carbonization of trisodium citrate dihydrate at the temperature of 500, 600, and 700 °C for 2 h with the heating rate of 2 °C min^−1^ under an argon atmosphere. The products were immersed in the dilute hydrochloric acid and washed repeatedly with DI water, and then vacuum freeze-drying for backup.

The synthesis of the PB sample was according to previously reported methods [29]. In particular, 1 mmol K_4_Fe(CN)_6_ was dissolved into 160 mL deionized water to form A solution, and 2 mmol FeCl_3_ was dissolved into 40 mL deionized water to form B solution. Then, the B solution is added dropwise to the A solution. The mixed solution was continued to be stirred for 2 h and then aged for 24 h after the A and B solutions are evenly mixed. Finally, the aged dark blue precipitate is centrifuged to obtain the PB products.

### Characterization

The morphology of the OFGC-500/600/700 was observed by scanning electron microscope (SEM, Ultra 55, Zeiss, Germany), Transmission electron microscope (TEM, Titan G2 60-300), and energy dispersive X-ray spectrometry (EDX) mapping. The chemical bonds were analyzed by X-ray photoelectron spectroscopy (XPS, ESCALAB 250Xi). Raman spectra were collected by Renishaw Raman Spectroscopy with a 633 nm laser as a source. The crystal phases were identified by X-ray diffraction (XRD, ULTIMA-3, Rigaku, Japan). The NOVA 1000e was used to measure the specific surface area of the OFGC-500/600/700 samples. In situ electrochemical impedance spectroscopy (EIS) was tested by the electrochemical workstation (IVIUM-VERTEX. C, Netherlands) with the frequency range from 0.01 to 100 kHz at a current density of 100 mA g^−1^. The cyclic voltammogram (CV) and the ex-situ EIS were measured by Electrochemical Workstation (CHI 660E, CHENHUA, Shanghai). And all electrochemical performances were tested by the Neware battery testing system (BTSCT-3008-TC).

### Electrochemical Measurements

The anode electrode was prepared with the active materials (OFGC-500/600/700), Ketjen black, and CMC (mass ratio of 7:2:1) dissolved into the H_2_O and ethanol (weight ratio of 4:1). The slurry after mixing for 8 h coated on the Cu foils, and dried at 80 °C in a vacuum overnight. The average active substance loading of each electrode is 0.6–1.0 mg. The cathode electrode was fabricated by dispersing the PB, Super P, and PVDF with a weight ratio of 6:3:1. The cathode collector is Al foils and the following steps are similar to the anode electrodes. All the 2032-type coin cells were assembled in the glovebox (H_2_O < 0.01, O_2_ < 0.01) within the Ar atmosphere. The electrolyte used in this paper is 5 M KFSI in DME, and the separator is Whatman glass fibers. Notably, the OFGC-600 anode and the PB cathode were pre-potassiation for 5 cycles before the full cells were assembled. The capacity and energy/power density of all full cells were calculated according to the total mass of the anode and cathode.

## Results and Discussion

### Morphology Control and Characterization of OFGC

**Fig. 1:**
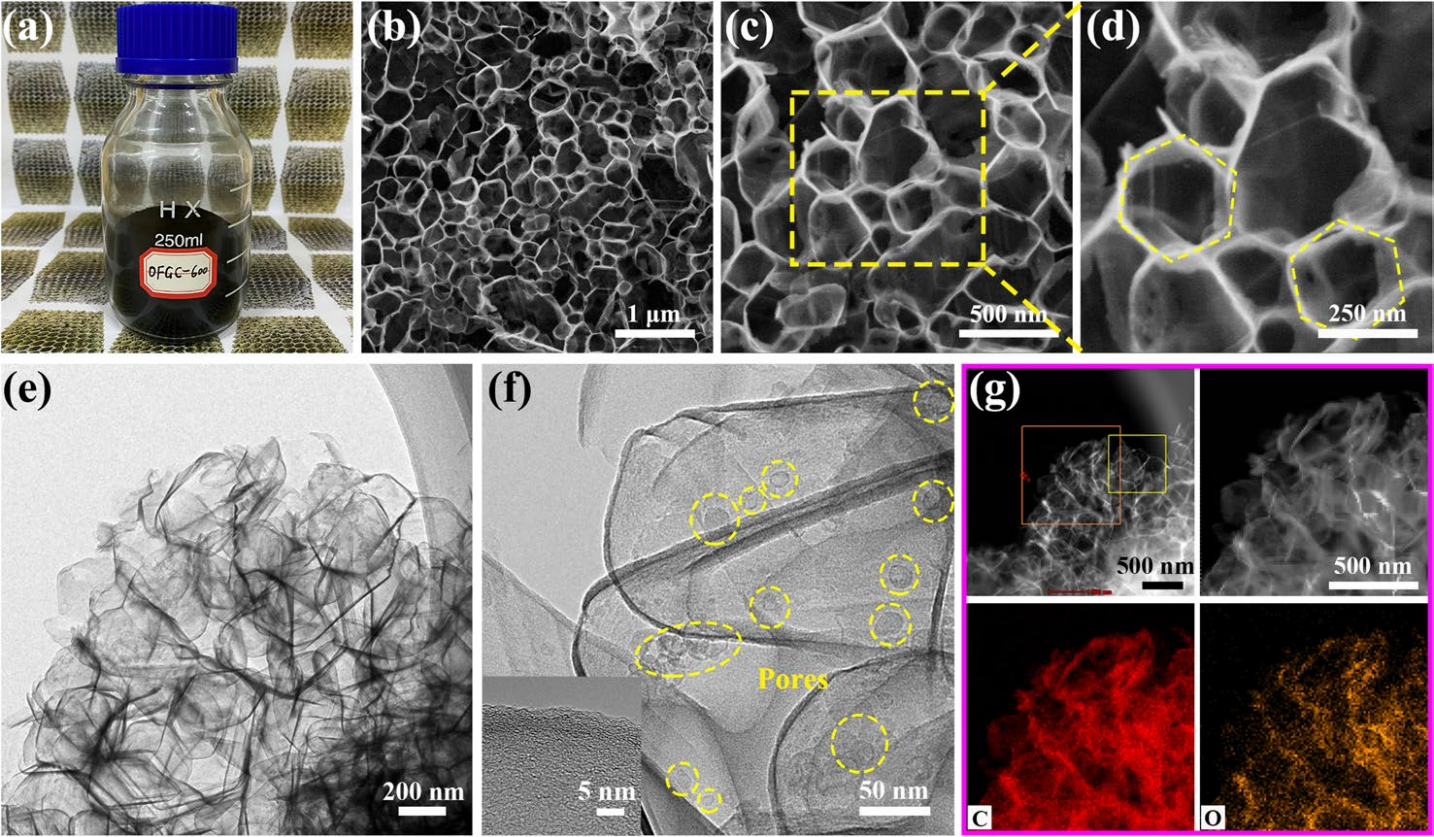
**a** Photograph of the final product of OFGC-600. SEM images of OFGC-600 at **b** the low magnification and **c**-**d** the high magnification, **e** TEM image. **f** The internal pore structure (circled in yellow) of OFGC-600 and the high-resolution TEM image of OFGC-600 (the lower-left illustration). **g** EDX elemental mapping of carbon and oxygen elements

The OFGC were synthesized facilely by one-step self-assembly and thermal polymerization of trisodium citrate dihydrate compound. The carbon source and oxygen source are derived from the low-cost organic reagent trisodium citrate dihydrate. A simple fabrication process of OFGC is illustrated in Fig. S1. Firstly, the precursor of trisodium citrate dihydrate was carbonized in an argon atmosphere at 500/600/700 ℃ to form the sodium carbonate/carbon composite. In this, sodium carbonate can act as a catalyst and template to form the honeycomb-like carbon structure. After diluting hydrochloric acid etching, the honeycomb-like OFGC named OFGC-500, OFGC-600, and OFGC-700 were synthesized at different heat treatment temperatures. All OFGC samples are cross-linked by multilayer nanosheets with porous structures, forming a continuous conductive honeycomb-like network. This honeycomb-like structure should be attributed to the unique template action of uniform Na_2_CO_3_ particles. Significantly, the interconnected nanosheets can not only expose more oxygen-containing functional groups but also promote electrolyte penetration, shorten ion diffusion distance and accelerate K^+^ transfer. More importantly, the honeycomb-like has the characteristics of a strong bearing structure, which can well prevent the collapse of the structure caused by the multiple depotassiation/potassiation process. Notably, OFGC electrodes derived from the organic reagent sodium citrate can easily be prepared in batches in the laboratory as shown in Fig. 1a. This laid a foundation for the commercialization of OFGC in PIBs at an early date. From the SEM and TEM images of Figs. 1b-e and S2a-b, the desired honeycomb-like structure can be observed. This result is consistent with what we expected above. In addition, the SEM images of the OFGC-500/700 samples showed no difference from OFGC-600 in Figs. S3a-c and S3d-f. The uniform pore structure as shown in Fig. 1f accelerates the infiltration of the concentrated salt electrolyte (5 M KFSI in DME). The illustration of high-resolution TEM (HRTEM) in Fig. S2c and the lower-left corner of Fig. 1f shows the amorphous structure between the carbon lamellar layers, which has a good ability to store K^+^. The EDX mapping in Fig. 1g further confirms that the honeycomb-like structure is composed of carbon and oxygen elements derived from the carbonization process of trisodium citrate dihydrate. Considering the stable honeycomb-like structure, the electrode material may have superior long cycling performance and good cation storage capacity.

**Fig. 2:**
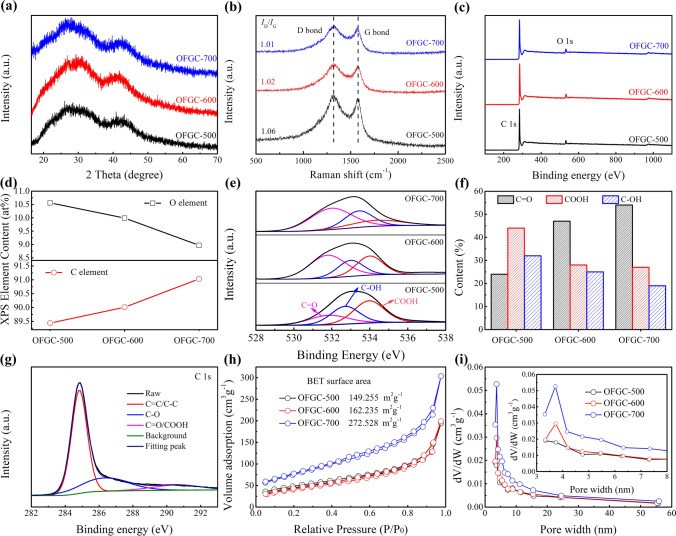
The physical and chemical properties of OFGC-500/600/700. **a** XRD patterns. **b** Raman spectra. **c** XPS survey spectra, and **d** element content of C and O. **e** High-resolution XPS spectra of O 1*s*, and **f** the content of C–OH, COOH, and C = O functional groups. **g** High-resolution XPS spectra of O 1*s* of OFGC-600. **h** Nitrogen adsorption–desorption isotherms of OFGC-500/600/700. **i** The total pore size distribution and the micropore size distribution (inset)

The physical, structure, and chemical properties of 3D honeycomb-like OFGC are further identified by XRD, Raman, and XPS. Figure 2a shows the XRD patterns of OFGC-500/600/700. The humpy peak at 24.6°–26.2° is (002) diffraction of disordered carbon, which is consistent with the HRTEM results. The broad (002) peak in the XRD pattern indicates that the graphitization degree of these three electrodes is relatively low. Raman spectra in Fig. 2b present two pre-dominant and overlapping peaks at 1346 cm^−1^ (D band) and 1596 cm^−1^ (G band). The enhancement of D band intensity indicates that OFGC is mainly composed of amorphous carbon (consistent with inset Fig. 1g) and has great structural defects. These defects may be caused by a large number of oxygen-containing functional groups, which may affect the physical and chemical properties of electrode materials, such as conductivity and edge effect, and thus play an important role in regulating the electrode interface and electrochemical properties. In addition, the ratio of *I*_D_/*I*_G_ directly reflects the degree of graphitization and defect degree. The *I*_D_/*I*_G_ value decreased as the synthesis temperature increased, indicating that the higher the graphitization degree, the fewer defects.

The surface characteristics and the quantity of the chemical composition of OFGC-500/600/700 were investigated by XPS analysis. The full spectrum of the XPS only contains C 1*s* and O 1*s* peaks in Fig. 2c, without other peaks appearing, indicating that OFGC only grafts oxygen elements. The content of carbon atom and oxygen atom in OFGC-500/600/700 were 89.5%, 90%, 91% and 10.5%, 10%, 9.0%, respectively. The atomic content of oxygen generally decreases with the increase in pyrolysis temperature, indicating that OFGC-500 has the most oxygen-containing functional groups and defects, and OFGC-700 has the least oxygen-containing functional groups and defects. In the high-resolution XPS spectrum of O 1*s* of OFGC-500/600/700 (Fig. 2e), three peaks centered at 286.1, 288.8, and 290.1 eV are overlapping and assigned to C = O carbonyl groups (O-I), C–OH hydroxylic groups or C–O–C ether groups (O-II), and COOH carboxyl groups (O-III), respectively [28, 30]. Notably, O-doping can improve the wettability of the electrolyte and introduces more active sites, leading to better reaction kinetics. This has been reported in the previous literature [27]. However, whether COOH and C = O functional groups can store K^+^ has not been studied. Figure 2f shows the histogram comparison of the content of functional groups of the OFGC-500/600/700 electrodes. For the high-resolution XPS spectrum of C 1*s* (in Fig. 2g) of OFGC-600, three peaks at 284.8, 286.1, and 289.8 eV correspond to the C–C/C = C, C–OH, and O = C-O/C = O bond, respectively [28, 31]. The chemical bond states of C 1*s* of OFGC-500/700 are not significantly different from those of OFGC-600 (Fig. S4).

The nitrogen adsorption/desorption isotherms of the OFGC-500/600/700 were obtained to evaluate the specific surface area and pore size distribution. As seen in Fig. 2h, all OFGC-500/600/700 display a typical type II isotherm with a high Brunauer–Emmett–Teller surface area of 149.255, 162.235, and 272.528 m^2^ g^−1^, respectively. Remarkably, the specific surface area of OFGC increases with the rise of temperature, which is consistent with the previous literature reports [27]. High nitrogen absorption at low relative pressure indicates that OFGC has high mesopores. The pore size distribution of OFGC-600 shows that the Barrett–Joyner–Halenda (BJH) desorption average mesopore diameter was 3.7 nm (Fig. 2i). This pore size may be due to the release of gases during carbonization. The mesopores can be acted as an ion buffer layer to promote the adsorption of K^+^ and speed up the depotassiation/potassiation process.

### Electrochemical Evaluation of the OFGC Anode

**Fig. 3:**
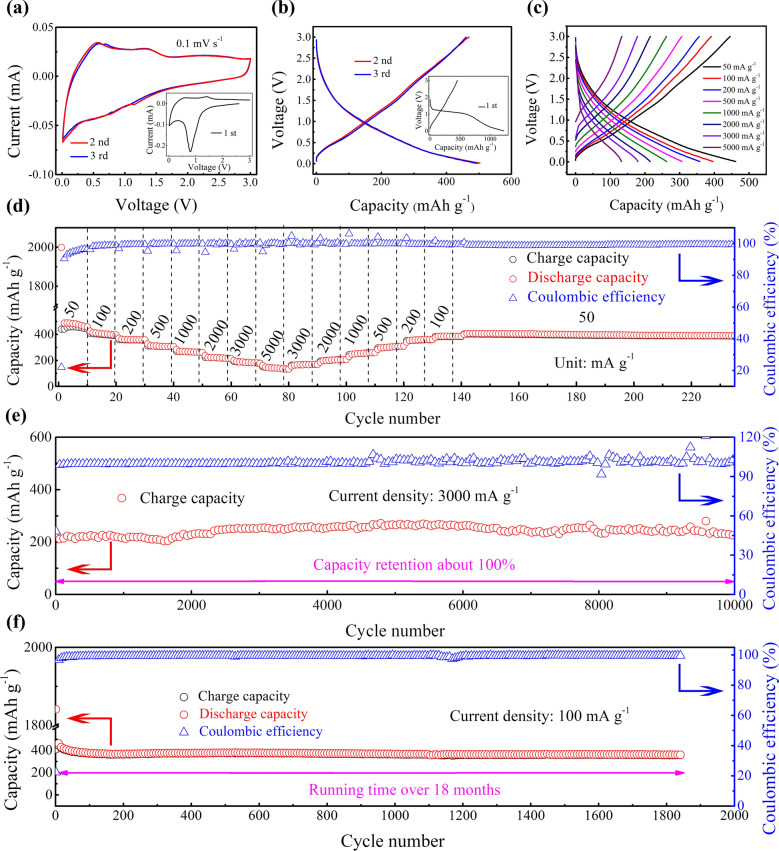
Cycle performance of as-synthesized OFGC-600 electrode. **a** CV curves of an as-fabricated OFGC-600 anode at the scanning rate of 0.1 mV s^−1^ (the inset is the first circle). **b** The galvanostatic charge/discharge curves for the first three cycles in the potential range of 0.01 − 3.0 V at 50 mA g^−1^. **c-d** Rate capability of OFGC-600 in the rate range from 50 to 5000 mA g^−1^. **e** Long-term cycle performance of OFGC-600at 3000 mA g^−1^ over 10,000 cycles. **f** Long-term cycle performance of OFGC-600 at a current density of 100 mA g^−1^ over 18 months

The OFGC electrode was applied as the anode for PIBs, and first using cyclic voltammetry (CV) and galvanostatic discharge/charge curves to evaluate the storage capacity of K^+^. Figure 3a shows the initial three CV curves of the OFGC-600 electrode with voltage window from 0.01 to 3.0 V at the scan rate of 0.1 mV s^−1^. Conspicuously, in the first cathodic scanning, a strong scanning peak appeared at 1.5–0.5 V and faded away in the following cycles, which may be due to the formation of SEI films and the decomposition of electrolytes in this process. Among them, the composition and formation process of the SEI films are crucial for the ultra-long cycle, which will be analyzed in detail in the following in situ EIS. In the subsequent anodic and cathodic scanning, the CV curves are highly overlapped, indicating that a highly reversible electrochemical reaction has occurred at or near the surface of the OFGC electrode. Notably, the anodic shows a weak peak at about 1.4 V, which may be related to the depotassiation process of COOH/C = O functional groups in the OFGC electrode. Figure 3b is the galvanostatic discharge/charge curves of the first three cycles, the OFGC-600 shows the charge/discharge capacity of 454/1224 mAh g^−1^ at 50 mA g^−1^. And the corresponding initial coulombic efficiency (ICE) is 37.1%. The lower ICE may be from the formation of SEI layers on the surface of OFGC caused by the higher specific surface area. This is common in carbon-based electrode materials [27, 32]. Fortunately, through the pre-potassiation process in the first dozen cycles, the CE can be greatly improved by more than 99%.

Figure 3c shows the discharge/charge curves under different current densities. Its shape is almost unchanged when the current density changes, which indicates that the reaction kinetics of K^+^ on OFGC-600 electrode material is significant. Because of this, the OFGC-600 electrode was tested for rate capability (Fig. 3d). When the current density increases from 50 mA g^−1^ to 5000 mA g^−1^, the reversible capacities of OFGC-600 are 456, 391, 355, 305, 260, 212, 177, and 134 mAh g^−1^, respectively. It is noteworthy that the OFGC-600 electrode can still achieve reversible capacities of 133, 168, 204, 258, 308, 357, 383, and 406 mAh g^−1^ when the current density decreases from a high current (5000 mA g^−1^) to a low (50 mA g^−1^). Such excellent rate performance is unique in the anode electrode of PIBs. More importantly, when returning to 50 mA g^−1^ after up to 14 current changes, it can still maintain a high capacity of 400 mAh g^−1^ and continue to circulate steadily for 100 cycles. To further clarify the relationship between the electrochemical performance and the structure and chemical composition of OFGC, the performance of the OFGC-500/600/700 electrode was compared at 500 mA g^−1^ exhibited in Fig. S5. The OFGC-500/600/700 all showed excellent cycle stability, while the capacity of OFGC-600 was slightly higher than the OFGC-500/700 electrode. This may be because the OFGC-600 electrode has the most suitable specific surface area and effective potassium storing oxygen functional group (COOH/C = O), which provides sufficient opportunities for the active sites to store more K^+^.

Furthermore, the cycling performance of OFGC-600 cycled at the high current of 3 000 mA g^−1^ for 10,000 cycles as shown in Fig. 3e. It is conspicuously that the OFGC-600 electrode still shows a charge capacity as high as 230 mAh g^−1^ with the capacity retention near 100%. Amazingly, the average coulombic efficiency around was 99% during 10,000 cycles. Moreover, as presented in Fig. 3f, the OFGC-600 electrode exhibits a cycle of up to 18 months and runs over 1800 cycles at a low current density of 100 mA g^−1^, and also maintains a high reversible capacity of 360 mAh g^−1^. The constant charge/discharge curves of different cycle numbers are shown in Fig. S6. Such long cycles are scarce in the anode materials of PIBs (Table S1). And the main reasons may be: (1) 3D stable honeycomb-like structure has continuous and interconnected large pore structure with large surface area and low mass density, which is an ideal scaffold for relieving volume expansion caused by multiple depotassiation/potassiation processes. The coarsely connected nanosheets with a microporous structure accelerate the infiltration of electrolyte and K^+^ transport. (2) Oxygen-rich doping exposes more active sites for absorbing K^+^ and improves the wettability of the electrolyte. Particularly, the COOH/C = O functional groups can reversibly store potassium and contribute to the increase in potassium capacity. (3) The COOH/C = O functional groups also regulate the composition (especially inorganic composition) of the SEI, which leads to highly conductive, intact, and robust SEI films. In short, all those (the sturdy honeycomb-like structure, reversible and efficiently COOH/C = O potassium storage sites, and the formation of robust SEI films) sets the stage for the OFGC-600 to exhibit high reversible capacity and ultra-stability at low or high current densities.

### Reversibility and Kinetics Analyses of OFGC Anode

**Fig. 4:**
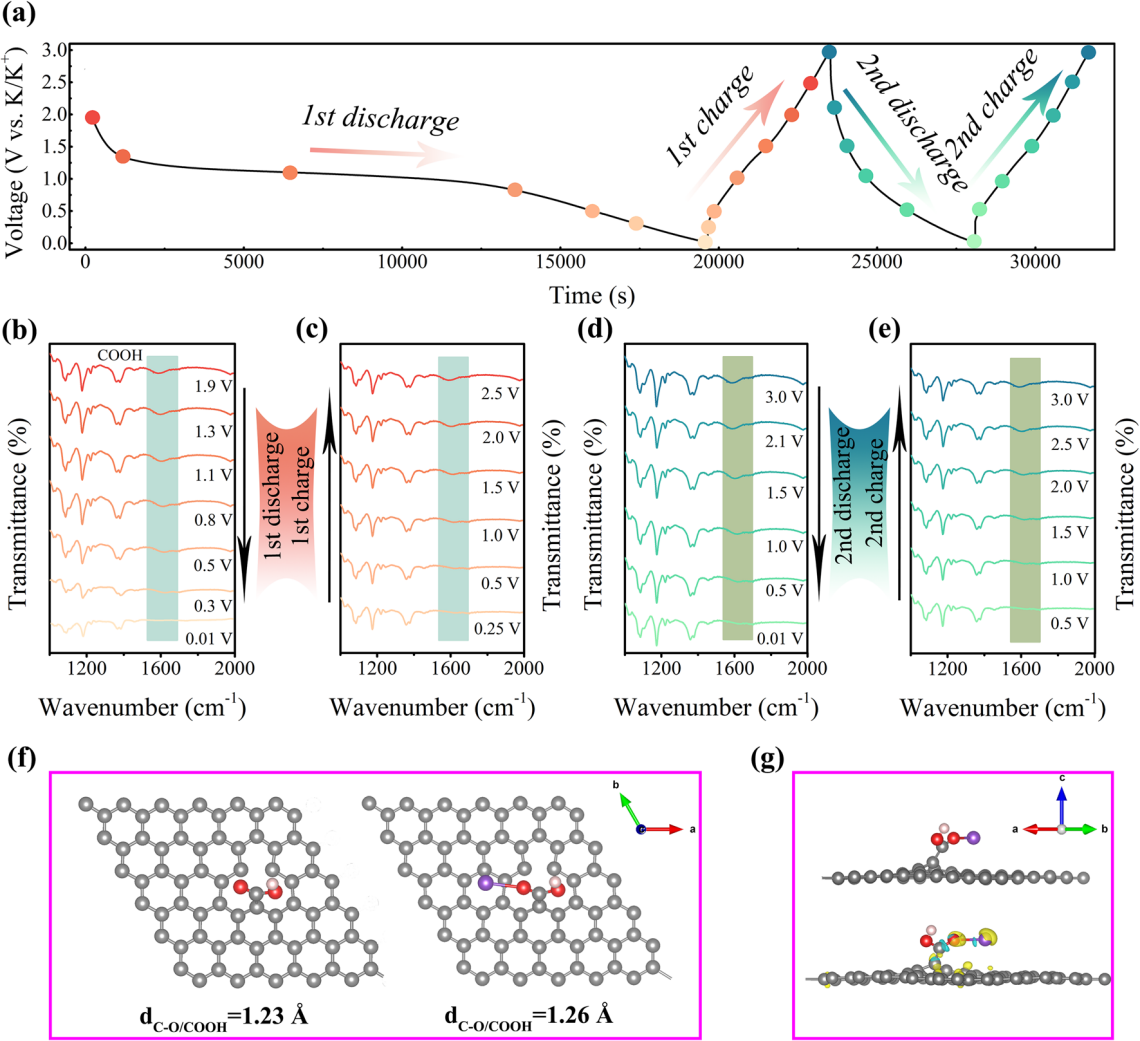
Analysis of potassium storage mechanism. **a** Galvanostatic charge/discharge profile of OFGC-600 during the in situ FT-IR measurement. **b**-**e** FT-IR spectra of OFGC-600 at different potentials during galvanostatic charge/discharge processes. **f** The optimized configurations of a single K atom adsorbed in graphene with COOH functional groups and **g** the charge density of graphene with COOH functional groups after adsorbing K ion. Yellow and blue areas represent increased and decreased electron density, respectively

To further explore the storage mechanism of COOH/C = O functional groups in OFGC-600 mentioned above in PIBs, the in situ Fourier transform infrared spectroscopy (FTIR) and density functional theory (DFT) calculations were performed. Figure 4a shows the charge/discharge curves of the voltage-time relationship. The discharge curve drops rapidly at 3.0–2.5 V, indicating that the main capacity contribution occurs at 2.5–0.01 V, while a relatively stable process occurs during charging. The changes in functional groups during the first discharge/charge, and second discharge/charge are shown in Fig. 4b-e, respectively. At the initial open-circuit voltage of 1.9 V, the distinct peak at 1680 cm^−1^ corresponds to the COOH functional group. Nevertheless, there are no obvious peaks corresponding to the C = O bond, which may be due to the weak peak strength that cannot be recognized by the instrument or the strong COOH peaks covering the peaks of the C = O (1720 cm^−1^) bond. It is worth noting that the position of the COOH peak moved slowly to the high wavenumber and disappeared completely when the initial discharge reached 0.01 V. Even more exciting, the COOH peaks gradually become apparent on the initial charging and return to the condition of open-circuit voltage at around 2.0 V. And the same phenomenon happens in the following cycle. We hypothesized that this might be a reversible reaction of COOH/C = O during the depotassiation/potassiation process. Therefore, we continue to use first-principles-based DFT calculations to explore the mechanism of potassium storage in the presence of K on COOH and C = O (specific calculation process in Supplementary S1.1). The optimal calculation model of 5 × 5 × 1 graphite super monomer was established, including COOH grafted graphene, C–OH grafted graphene, and C = O grafted graphene. Note that there are too many defects to be optimized in the calculation process, so only one defect grafted COOH and C = O functional group are constructed in the model. Figure S7 shows the optimum adsorption sites and corresponding adsorption energy (Δ*E*a) of K^+^ on the three functional groups. The Δ*E*a of K^+^ in COOH and C = O doped graphene layer are −2.76 and −2.80 eV, higher than that of the C–OH group (−2.32 eV). This result shows that COOH and C = O are more conducive to the rapid adsorption of K ions. In addition, the bond lengths of C = O before and after the interaction of K^+^ on COOH and C = O groups are shown in Figs. 4f and S8a. The carbon–oxygen double bond (λ) in the COOH and C = O groups was 1.23 Å, much lower than that of 1.26/1.28 Å after the simulated potassiation process. Those results indicate that the carbon–oxygen double bond is broken and combined with K during the potassiation process. Besides, we also simulated the differential charge density of K^+^ adsorption on COOH/C = O groups, as shown in Figs. 4g and S8b. It can be found that the electron density of the C = O double bond on COOH/C = O functional groups decreases after potassium adsorption, while the electron density between K–O increases, indicating that the electron is transferred from the C = O double bond to K–O, which proves once again that the C = O double bond becomes a single bond and combines with K to C-O-K. Moreover, the C = O double bond can be returned to 1.23 Å when the adsorption of potassium is removed. Combined with the above in situ FTIR results, the reaction mechanism of COOH and C = O during the potassiation and depotassiation process in OFGC may be as follows:

1 $${-}{\text{C = O + K}}^{ + } {\text{ + e}}^{ - } \leftrightarrow {-}{\text{C}}{-}{\text{O}}{-}{\text{K}}$$

2 $${\text{HO}}{-}{\text{C}} = {\text{O + K}}^{ + } {\text{ + e}}^{ - } \leftrightarrow {\text{HO}}{-}{\text{C}}{-}{\text{O}}{-}{\text{K}}$$

Gratifyingly, this reaction mechanism is reversible, which again explains the high capacities and superior long cycles of the OFGC-600 electrode in Fig. 3.

**Fig. 5:**
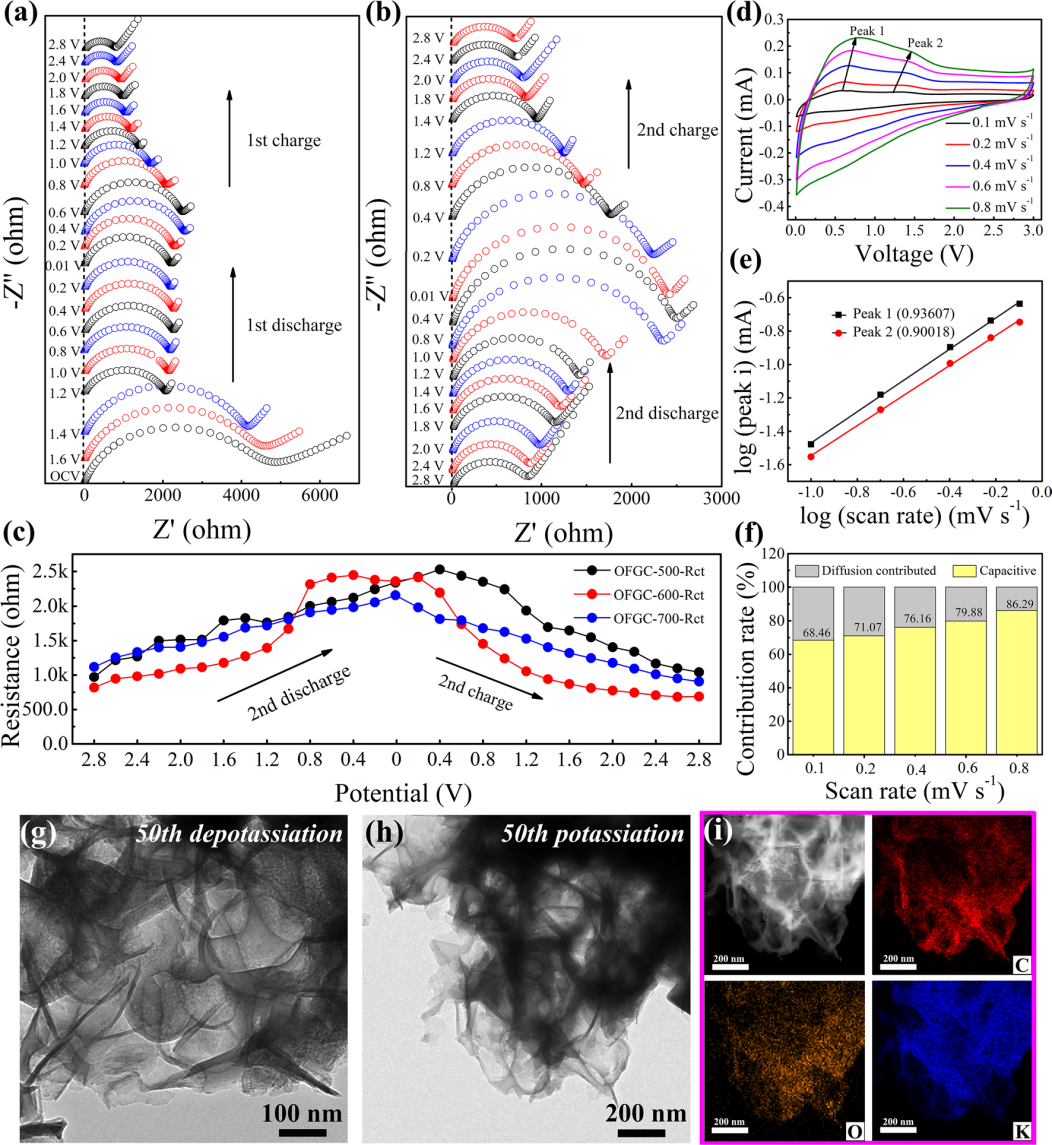
**a**-**b** Nyquist plots of OFGC-600 during charge and discharge for the first two cycles and **c** the charge transfer impedances (*R*_ct_) of OFGC-500/600/700 at the second cycle. **d** CV curves and **e** the *b* values plotted for the potential anodic peak. **f** Capacitive contribution under scan rates of 0.1, 0.2, 0.4, 0.6, and 0.8 mV s^−1^. **g**–**h** TEM images after 50th depotassiation and 50th potassiation. **i** EDX elemental mapping of C, O, and K elements after 50th potassiation

The in situ EIS are used to analyze the reaction dynamics and the SEI evolution of OFGC during the discharge and charge process. The high/medium frequency region in all Nyquist diagrams is an approximate semicircle, which represents the charge transfer resistance (*R*_*ct*_), in particular, indirectly reflects the resistance of the SEI film in some literature [33, 34]. Conversely, the slope of an oblique line in the low-frequency range represents the Warburg impedance of the ion diffusion [25]. The Nyquist diagram is fitted with the equivalent circuit model to analyze the changes of resistances (Fig. S9). In the initial discharge process of OFGC-600 shown in Fig. 5a, the *R*_ct_ of the open-circuit voltage (OCV) state is about twice that of the 1.2 V state. Obviously, the *R*_ct_ decreased significantly with the gradual embedding of K^+^ into the OFGC-600 electrode, indicating that a conductive SEI film was formed at this stage, which is consistent with the initial CV diagram in Fig. 3a. In addition, when the deep discharge continued to 0.01 V, the resistance of *R*_ct_ in the voltage range of 1.2–0.01 V was almost constant, manifesting that the metastable SEI film had been formed at high potential. In contrast, during the initial charge process, the *R*_ct_ decreased from 2251 Ω (0.2 V) to about 771 Ω (2.8 V), indicating faster charge transfer and reaction kinetics during the depotassiation process. However, the resistance gradually rises from 818 Ω (2.8 V) to around 2300 Ω (0.01 V) during the second discharge process, and again drops to 648 Ω when charging to 3.0 V. The regular changes of *R*_ct_ with the discharge/charging process at the second cycle are also shown in OFGC-500/700, which again proves the high reversibility of the OFGC anode (Fig. S10a-b). In addition, the statistics of *R*_ct_ of OFGC-500/600/700 in the second cycle are shown in Fig. 5c. The *R*_ct_ of OFGC-600 is lower than OFGC-500/700 in both discharge (2.8 to 0.8 V) and charge (0.6 to 2.8 V) processes. This suggests that OFGC-600 has a faster charge transfer resistance than the OFGC-500/700 electrode. However, the *R*_ct_ of the OFGC-500/600/700 electrode is increased in the voltage range from 0.8 to 0.01 V (discharge process) and 0.01 to 0.6 V (charge process). This may be because C = O/COOH functional groups exposed in OFGC form metastable SEI films in the initial circle, resulting in reconstruction of the electrode structure when K^+^ is embedded, which blocks the transport of K^+^ at low potential. In addition, C = O and COOH functional groups can promote the formation of inorganic components of SEI films, which has been reported in our previous paper [25]. To account for this stable inorganic SEI membrane in the OFGC-600 electrode, we also tested the impedance of the original, 5th, and 500th cycles after full discharge (Fig. S11). Not surprisingly, the value of *R*_ct_ of the 5th (about 2350 Ω) was almost the same as that of the 1st (about 2250 Ω), indicating that a stable SEI was basically formed during the first five cycles. In addition, there was no significant difference between the value of *R*_ct_ of 500th (about 2700 Ω) and the 5th (about 2350 Ω), indicating that the SEI film existed stably after a long cycle, which again proved the stable structure of the OFGC-600 electrode.

Inspired by the excellent rate performance of OFGC-600, the reaction kinetics were measured through mathematical analysis of anode and cathode at different scan rates from 0.1 to 0.8 mV s^−1^ as exhibited in Fig. 5d. From the fitted logarithmic (ν) -logarithmic (*i*) diagram (formula and calculation process in Supplementary S1.2), the b values of the anodic peak of OFGC-600 are calculated to be 0.93 (peak 1) and 0.90 (peak 2) shown in Fig. 5e. This result indicates that diffusion behavior and capacitance behavior coexist in the OFGC-600 electrode, and capacitance behavior is dominant. In addition, when the scan rate test was performed at 0.1, 0.2, 0.4, 0.6, and 0.8 mV s^−1^, the calculated pseudo-capacitive contribution corresponds to 68.46%, 71.07%, 76.16%, 79.88%, and 86.29%, respectively (statistical histograms are shown in Fig. 5f. Figure S12 is the CV curves of capacitive current compared with the total measured current at 0.6 mV s^−1^). The significant capacitance contribution indicates that the near-surface region rapid response at low or high scan rates, which also explains the excellent rate performance in Fig. 3c-d. Similarly, the stable structure has been confirmed by the ex-situ TEM images after the 50th depotassiation (Figs. 5g and S13a-d)/potassiation (Figs. 5h and S13e-h) processes**.** The honeycomb-like structure of the OFGC-600 electrode is still maintained complete after the 50th potassiation and depotassiation processes. This result shows that the honeycomb structure is more stable and alleviates the volume expansion caused by large K^+^ de/insert. Obviously, the EDX profile in Fig. 5i shows that the K element can completely cover the O element, indicating that potassium ions and COOH/C = O functional groups can fully react after being discharged to 0.01 V. On the contrary, the distribution of K elements becomes blurred when fully charged to 3.0 V (Fig. S14), but still exists a small amount of K, which may be because the COOH/C = O functional group participates in the regulation of the composition of SEI with K elements and forms a stable inorganic (K_2_CO_3_) SEI film.

### Electrochemical Evaluation of Full Cell and Practical Application

**Fig. 6:**
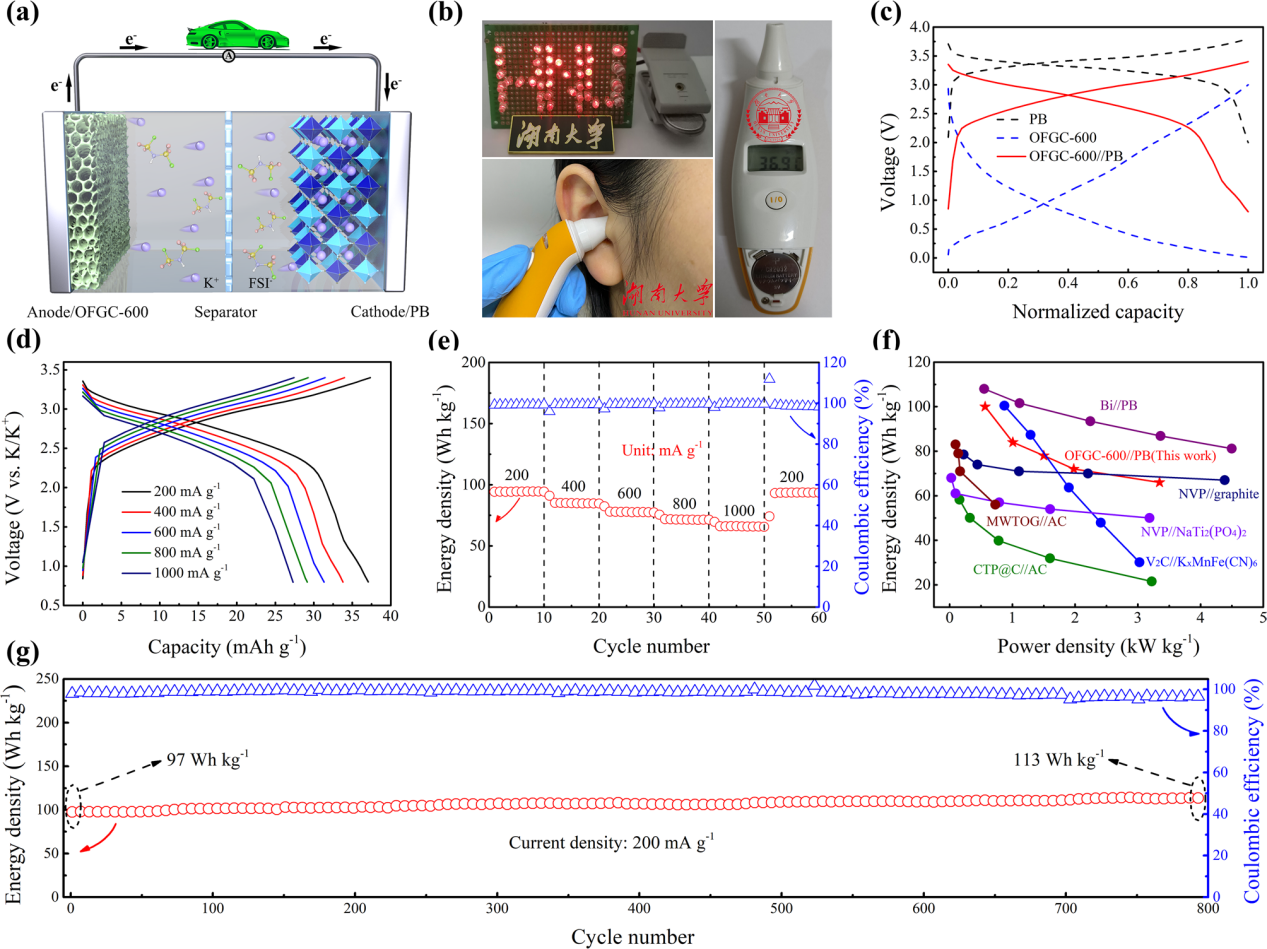
**a** Schematic illustration of the discharge mechanism of the OFGC-600//PB full cell. **b** The devices of an LED bulb with “HNU” and the ear thermometer driven by the full cells. Electrochemical performances of the full cells. **c** The charge/discharge curves of PB, OFGC-600, and OFGC-600//PB. **d**–**e** The rate capability at different current densities from 200 to 800 mA g^−1^. **f** Ragone plot of OFGC-600//PB full cell compared with the reported articles. **g** Cycling performance at a current density of 200 mA g^−1^

To study the energy density of OFGC-600 and its potential in practical applications, a full battery was assembled with OFGC-600 as the anode electrode, PB as the cathode, and 5 M KFSI in DME as electrolyte (the full battery is abbreviated as OFGC-600//PB). The reaction diagram of the full battery is shown in Fig. 6a. Normally, K^+^ escapes from the OFGC-600 anode and migrates to the cathode PB during the discharge process, while the corresponding electrons flow from the anode to the cathode through an external circuit. The charge process is the opposite process. In Fig. 6b, a fully charged full battery can successfully drive electronic devices such as LEDs (the operating voltage is 1.9–2.2 V) with “HNU” shaped and ear thermometers, which indicates that OFGC-600//PB full cells have good applications in life. The full cells also detected energy/power density and cycle performance with the voltage range of 0.8–3.2 V. The voltage window of OFGC-600//PB was determined according to the voltage of OFGC-600 and PB half cells. The charge/discharge profiles of anode/cathode half cells and full cells are shown in Fig. 6c. Sloping charge/discharge curves, indicating different processes of K^+^ interaction with cathode/anode. The OFGC-600//PB full cells manifest excellent rate performance. The profiles of OFGC-600//PB at different current densities ranging from 200 to 1000 mA g^−1^ remain consistent as exhibited in Fig. 6d, indicating that the full cells have fast potassium storage behavior. The OFGC-600//PB full cell also showed high energy density and power density. For example, the full cell delivers energy density of 94, 84, 77, 71, and 65 Wh Kg^−1^ (all calculated according to the total weight of cathode and anode) at the current density of 200, 400, 600, 800, and 1000 mA g^−1^, respectively (Fig. 6e). The corresponding capacity at various current densities is presented in Fig. S15. Notably, by adjusting the current density from low to high 5 times, the full cells can still up to a high energy density of 100 Wh Kg^−1^ when returning to the low current density of 200 mA g^−1^, showing an amazing rate performance. In addition, compared with previous research on PIBs [35], potassium-ion hybrid capacitors (PIHCs) [36, 37], sodium-ion batteries (NIBs) [38, 39], and sodium-ion capacitors [40], the OFGC-600//PB full cells still exhibit superior power density shown in Ragone plots in Fig. 6f and Table S2. In particular, in Fig. 6g, the full cell shows an energy density of 113 Wh kg^−1^ after operating 800 cycles at 200 mA g^−1^, exhibiting excellent cycle performance (Fig. S16 is the corresponding capacity–cycle diagram).

## Conclusion

In summary, a 3D honeycomb-like carbon grafted with effectively active oxygen-rich functional groups was synthesized by self-assembly pyrolysis of sodium citrate. The stable 3D honeycomb-like structure with continuous and interconnected large pore structure alleviates the volume expansion caused by the insertion of K^+^ into OFGC. The grafting of the COOH/C = O functional group improves the specific capacity of the OFGC electrode by forming C-O-K compounds with potassium, and this reaction is highly reversible during the charge/discharge process. In addition, COOH/C = O functional groups also can participate in the regulation of SEI components to form robust inorganic SEI membranes. Consequently, the battery shows a super stable cycle over 1800 times with a reversible capacity retention of 360 mAh g^−1^ at 100 mA g^−1^, corresponding to a runtime of up to 18 months. The full cells assembled by OFGC-600 anode and PB cathode also delivered excellent performance, especially its higher energy density could be up to 113 Wh kg^−1^, which can get the LED lamp light up and the ear thermometer running. This work may provide a new thought for the optimization of oxygen-containing functional groups in electrode engineering, and demonstrate the application potential of anode of oxygen-rich functional groups in PIBs.

## Supplementary Information

Below is the link to the electronic supplementary material.

Supplementary file1 (PDF 1817 KB)
