# Supplementary material for: Reversible Oxygen-Rich Functional Groups Grafted 3D Honeycomb-Like Carbon Anode for Super-Long Potassium Ion Batteries
Source: Nanomicro Lett. 2022 Jul 21;14:146. doi: 10.1007/s40820-022-00892-8 (PMC9304482; doi:10.1007/s40820-022-00892-8)
Supplement: Supplementary file 1 — Supplementary file1 (PDF 1817 KB) [file 40820_2022_892_MOESM1_ESM.pdf]

Supporting Information for

Reversible Oxygen-Rich Functional Groups Grafted 3D Honeycomb-Like Carbon Anode for Super-Long Potassium Ion Batteries

Na Cheng[1], Wang Zhou[2], Jilei Liu[2,]*, Zhigang Liu[1,]*, Bingan Lu[3]

[1] School of Chemistry and Chemical Engineering, Hunan University, Changsha 410082, P. R. China

[2] School of Materials Science and Engineering, Hunan University, Changsha 410082, P. R. China

[3] School of Physics and Electronics, Hunan University, Changsha 410082, P. R. China

* Corresponding authors. E-mail: liuzhigang@hnu.edu.cn (Zhigang Liu); liujilei@hnu.edu.cn (Jilei Liu)

S1 Supplementary Text

S1.1 Computational Details

First-principles computations based on density functional theory (DFT) were implemented in the Vienna Ab initio simulation package (VASP) [1]. The generalized gradient approximation (GGA) involving Perdew-Burke-Ernzerhof (PBE) [2] functional was used for calculating the exchange-correlation energy. A 400 eV cut-off energy was adopted for the plane-wave basis set in conjunction with the projector augmented wave (PAW) [3]. The energy and force convergence was set to be 1×10^{-4} and 2×10^{-2} eV respectively. The Brillouin zone was sampled using the Monkhorst-Pack scheme, K-points were generated by VASPkit [4], and the recommended value is 0.04 ($2\pi\times0.04$ Å^{-1}). The adsorbed energy (E_b) of K ions is defined as : $E_b = E_{total} - E_G - E_K$, where E_{total} denotes the DFT total energy of K ion absorbed on the Graphene, E_K is the energy of K atoms and E_G is the total energy of Graphene.

S1.2 Capacitive Contribution in OFGC-600

Through mathematical analysis of anode and cathode at different scan rates, the storage mechanism of K^+ in OFGC-600 can be determined. The value of b can be determined according to the relationship between scan rate (v, mV s^{-1}) and peak current (i, mA) (Eq. S1):

$$i = av^b \qquad (S1)$$

When the value of b is 0.5, it indicates limited diffusion. And if the value of b is 1, it implies an activation polarization reaction. This reaction limitation process includes capacitive behavior but is not limited to surface capacitance.

The quantitative calculation of the proportion of diffusion behavior and activation polarization behavior contribution to the overall capacity of the OFGC-600 electrode can be evaluated based on the following Eq. (S2):

$$i = k_1 v + k_2 v^{1/2} \qquad (S2)$$

Where $k_1 v$ is the contribution of the capacitance-controlled process, and $k_2 v^{1/2}$ is the contribution of the ionic diffusion-controlled process.

S2 Supplementary Figures and Tables

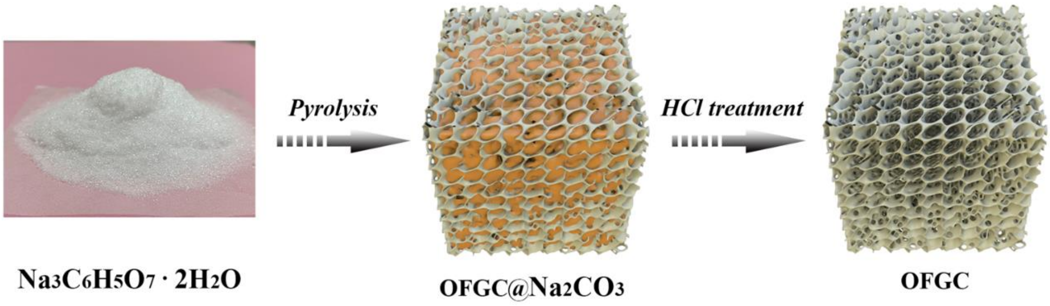

Fig. S1 The formation process diagram of the 3D honeycomb-like OFGC sample

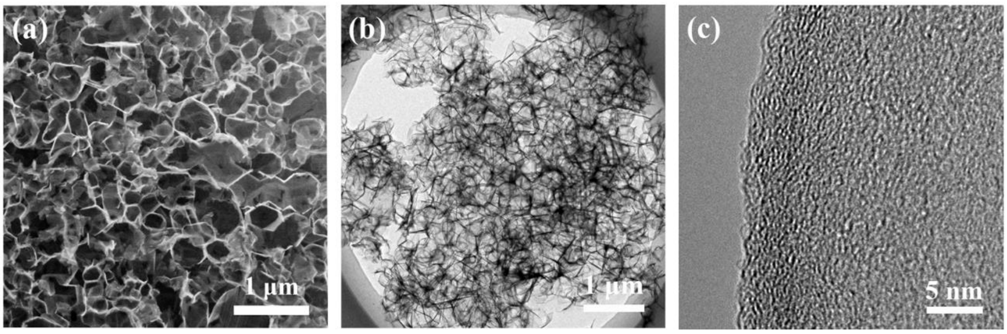

Fig. S2 a) SEM images of OFGC-600. **b**) TEM image at 1μm. **c**) High-resolution TEM image of OFGC-600

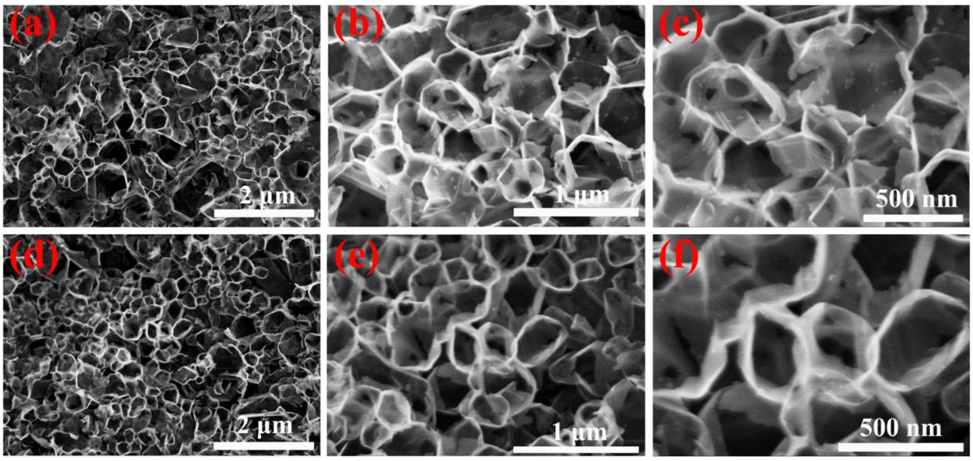

Fig. S3 a-c) SEM images of OFGC-500. **d-f**) SEM images of OFGC-700

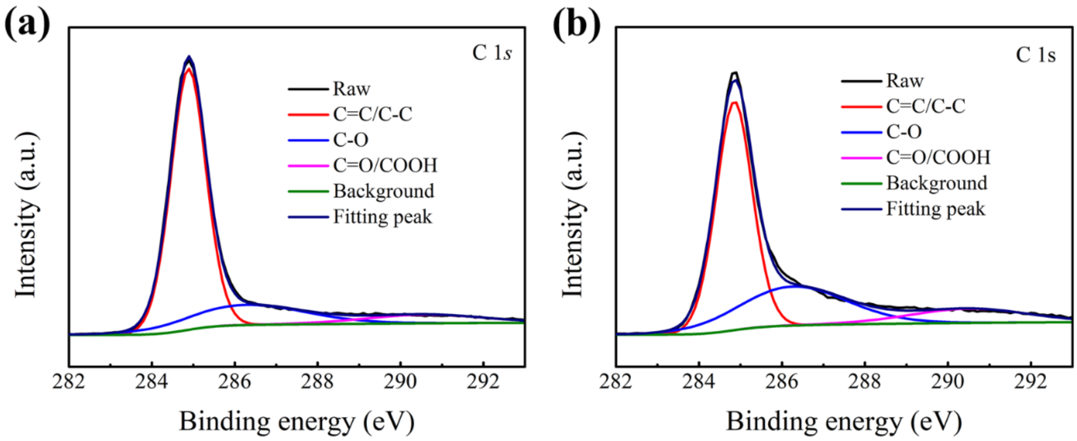

Fig. S4. High-resolution XPS spectra of C 1s: **a**) OFGC-500, **b**) OFGC-700

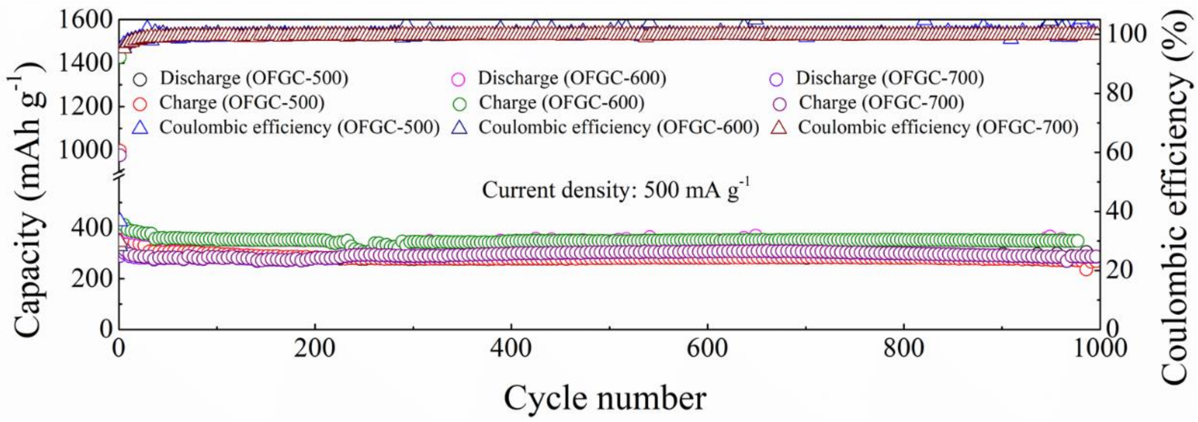

Fig. S5 Cycle performance of OFGC-600 compared with OFGC -500/700 at 500 mA g^{-1}

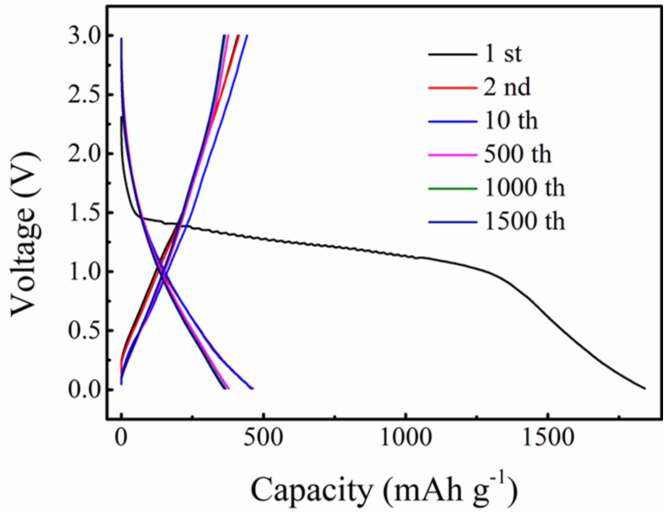

Fig. S6 Galvanostatic discharge/charge profiles of OFGC-600 at 100 mA g^{-1} at 1st, 2nd, 10th, 500th, 1000th, and 1500th cycles

Table S1 Comparison of electrochemical performances of OFGC-600 half cell with those of the previously reported carbon-based materials

Materials	Current density	Capacity maintain (cycle number)	Ref.
RPCNS	50 mA g^{-1}<br>1000 mA g^{-1}	346 mAh g^{-1} (360 cycles)<br>243 mAh g^{-1} (2000 cycles)	5
NOPC@G	111.6 mA g^{-1}<br>1116 mA g^{-1}	287 mAh g^{-1} (100 cycles)<br>160 mAh g^{-1} (500 cycles)	6
S/O-PCMs	50 mA g^{-1}<br>1000 mA g^{-1}	226 mAh g^{-1} (100 cycles)<br>108.4 mAh g^{-1} (2000 cycles)	7
NMC	100 mA g^{-1}<br>1000 mA g^{-1}	251.2 mAh g^{-1} (450 cycles)<br>101.4 mAh g^{-1} (1000 cycles)	8
N/P-HPCB	100 mA g^{-1}<br>2000 mA g^{-1}	458.3 mAh g^{-1} (100 cycles)<br>205.2 mAh g^{-1} (1000 cycles)	9
S-NC	2000 mA g^{-1}	141 mAh g^{-1} (3000 cycles)	10

NPC	50 mA g^{-1}<br>500 mA g^{-1}	296.8 mAh g^{-1} (100 cycles)<br>121.3 mAh g^{-1} (1000 cycles)	11
NPC	100 mA g^{-1}<br>1000 mA g^{-1}	384.2 mAh g^{-1} (500 cycles)<br>226.1 mAh g^{-1} (1000 cycles)	12
MCOs	100 mA g^{-1}<br>2000 mA g^{-1}	240 mAh g^{-1} (100 cycles)<br>80 mAh g^{-1} (3000 cycles)	13
NSHCCs	50 mA g^{-1}<br>1000 mA g^{-1}	310 mAh g^{-1} (620 cycles/9 months)<br>145 mAh g^{-1} (3000 cycles)	14
BCCs	100 mA g^{-1}<br>500 mA g^{-1}	302 mAh g^{-1} (1800 cycles/15 months)<br>226 mAh g^{-1} (2100 cycles)	15
OFGC	100 mA g^{-1}<br>3000 mA g^{-1}	360 mAh g^{-1} (1800 cycles/18 months)<br>229 mAh g^{-1} (10000 cycles)	This work

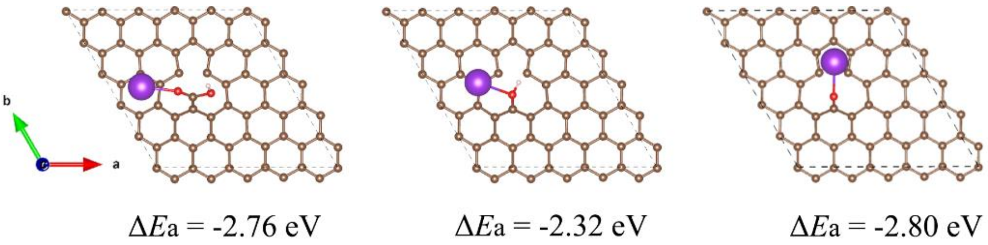

Fig. S7 The optimized configurations of a single K atom adsorbed in graphene with three functional groups (COOH, C-OH, and C=O)

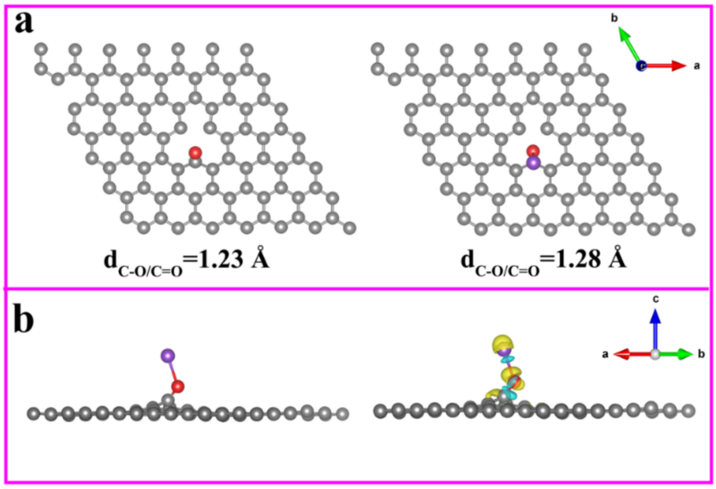

Fig. S8 a) The optimized configurations of a single K atom adsorbed in graphene with C=O functional groups and **b**) the charge density of graphene with C=O functional groups after adsorbing K ion. Yellow and blue areas represent increased and decreased electron density, respectively

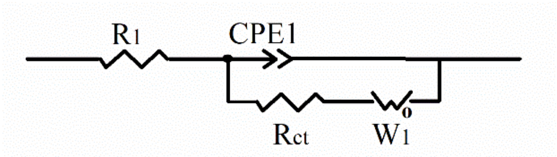

Fig. S9 The equivalent circuit model of the Nyquist diagram of OFGC-500/600/700 electrode

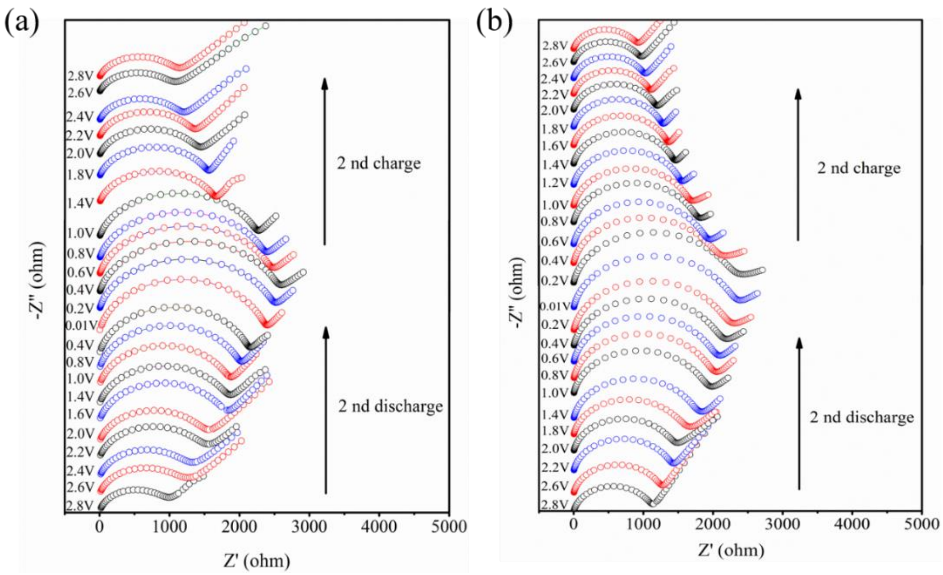

Fig. S10 Nyquist plots of **a**) OFGC-500 and **b**) OFGC-700 at different potentials during the second discharge/charge process

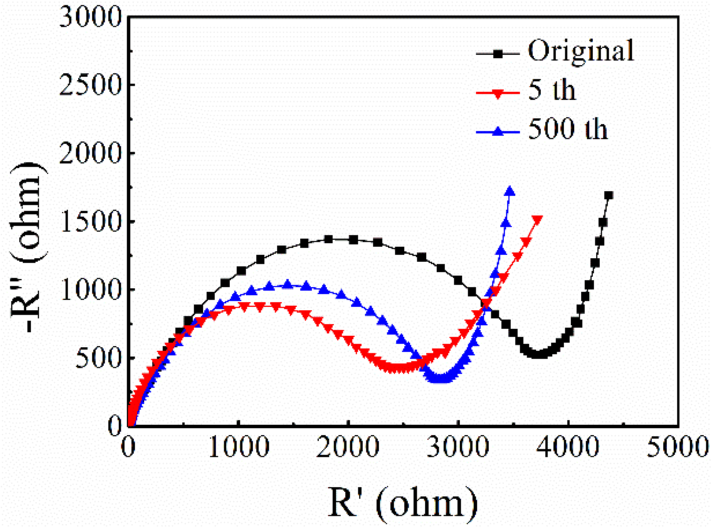

Fig. S11 Nyquist diagram of OFGC-600 in different charging/discharging processes (original, 5th, and 500th)

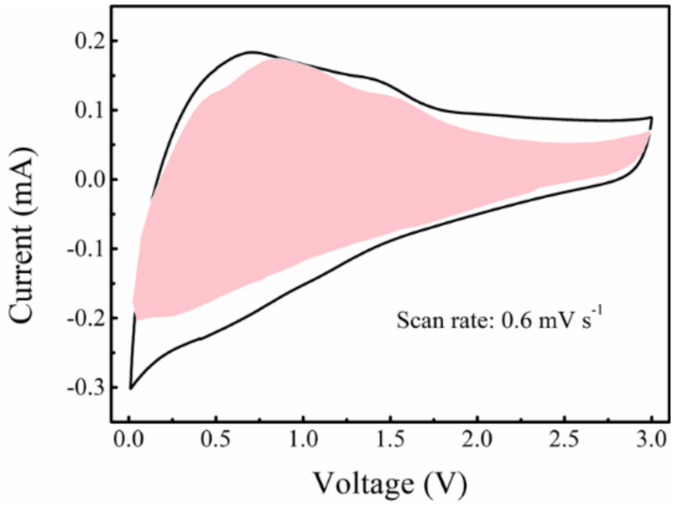

Fig. S12 Contribution of the capacitive at the scan rate of 0.6 mV s^{-1}

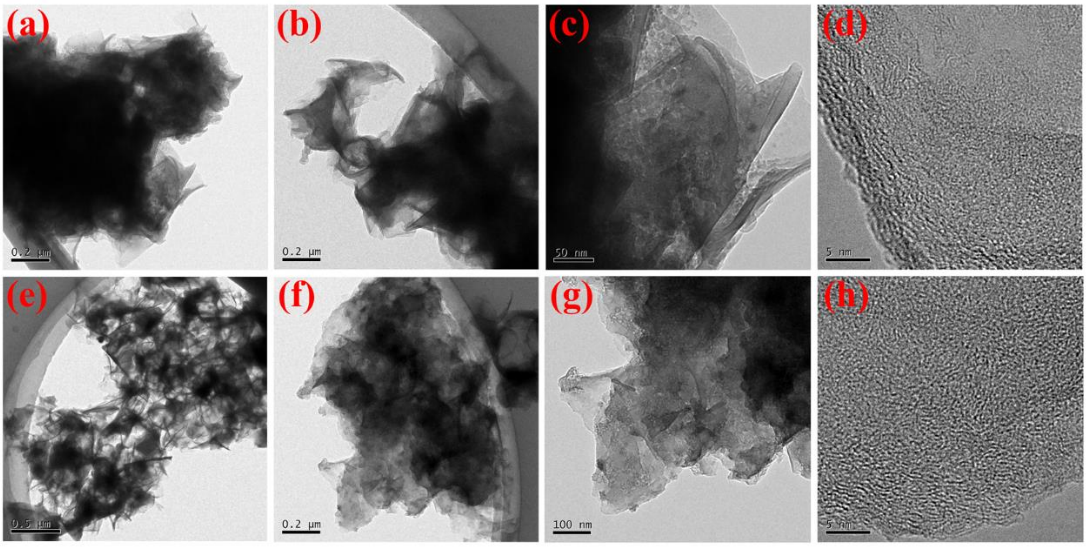

Fig. S13 TEM images and High-resolution TEM image of OFGC-600 after 50 cycles. **a-d**) discharged to 0.01 V, **e-h**) charged to 3.0 V

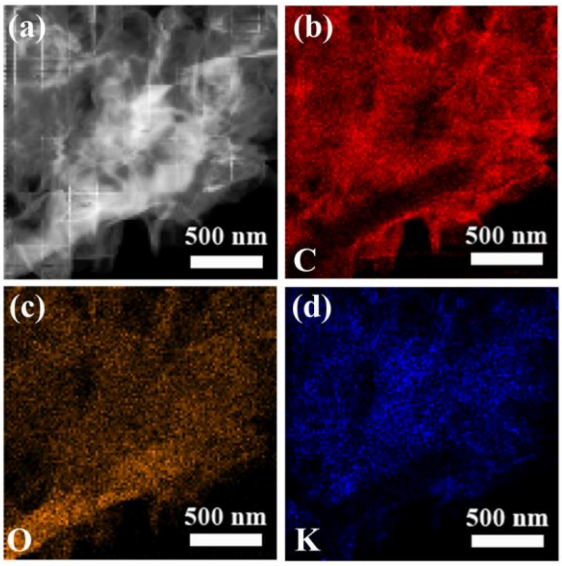

Fig. S14 EDS elemental mapping images after 50 cycles at charged to 3.0 V state

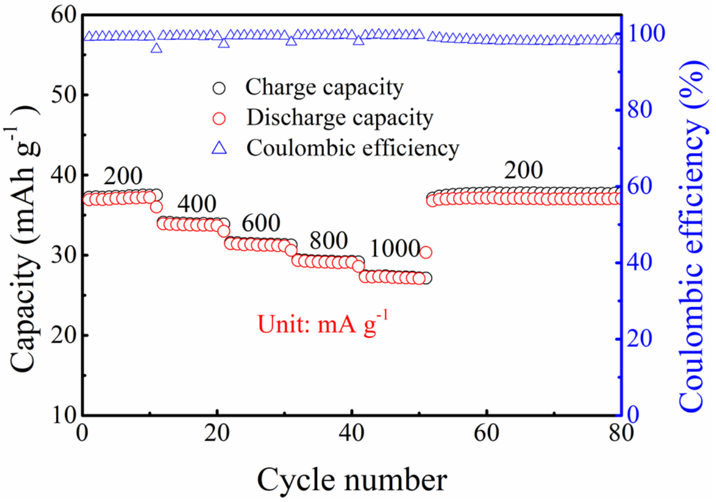

Fig. S15 Rate performance of OFGC-600//PB full cell at the current density of 200, 400, 600, 800, and 1000 mA g^{-1}

Table S2 Comparison of electrochemical performances of OFGC-600//PB full cell with those of the previously reported full batteries with PB or PBAs as the cathode

Full cell	Voltage (V)	Energy density/ current density	Power density/ current density	Cycle number/ current density/ retention (%)	Ref.
Bi//PB	0.8-3.6	108/800	4500/1600	350/800/63.3	16
K-V_2C//$K_xMnFe(CN)_6$	0.01-4.6	100.4/700	872/800	200/1000/95	17
Super P//KPBNPs	1.0-3.8	–	–	50/~50/90	18
WS_2//$K_{1.98}Mn[Fe(CN)_6]_{0.92}$	2.0-4.0	92/100	–	10/100/80	19
S/N-CNFAs//KPB	2.0-4.2	–	–	60/~33/91	20
NCNF//KPB	2.0-4.2	–	–	30/~40/97	21
N, P-VG@CC//KPB	2.0-4.2	232/50	4000/2000	150/50/85.9	22
Fe_7S_8@CNT@3DFG//KPB	0.5-3.2	120/100	–	65/100/100	23
OFGC-600//PB	0.8-3.4	113/200	3346/1000	800/200/100	This work

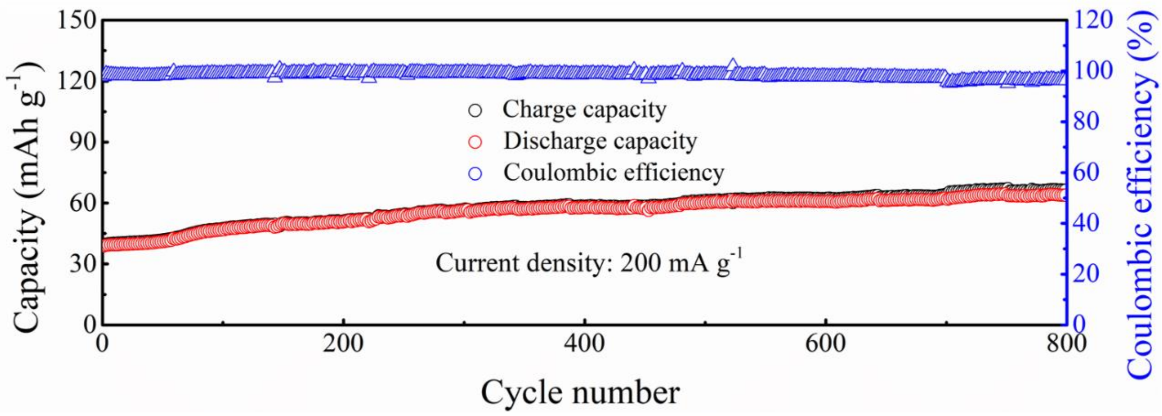

Fig. S16 Cycle performance of OFGC-600//PB full cell at current density 200 mA g^{-1}

Supplementary References

[1] G. Kresse, J. Furthmuller, Efficient iterative schemes for ab initio total-energy calculations using a plane-wave basis set. Phys. Rev. B **54**(16), 11169-11186 (1996). https://doi.org/10.1103/physrevb.54.11169

[2] J.P. Perdew, K. Burke, M. Ernzerhof, Generalized gradient approximation made simple. Phys. Rev. Lett. **77**(18), 3865-3868 (1996). https://doi.org/10.1103/physrevlett.77.3865

[3] G. Kresse, D. Joubert, From ultrasoft pseudopotentials to the projector augmented wave method. Phys. Rev. B **59**(3), 1758-1775 (1999). https://doi.org/10.1103/PhysRevB.59.1758

[4] V. Wang, N. Xu, J.C. Liu, G. Tang, W.T. Geng, Vaspkit: a user-friendly interface facilitating high-throughput computing and analysis using vasp code. Comput. Phys. Commun. **267**, 108033 (2021). https://doi.org/10.1016/j.cpc.2021.108033

[5] H. Deng, L. Wang, S. Li, M. Zhang, T. Wang et al., Radial pores in nitrogen/oxygen dual-doped carbon nanospheres anode boost high-power and ultrastable potassium-ion

batteries. Adv. Funct. Mater. **31**(51), 2107246 (2021). https://doi.org/10.1002/adfm.202107246

[6] Y. Zhu, M. Wang, Y. Zhang, R. Wang, Y. Zhang et al., Nitrogen/oxygen dual-doped hierarchically porous carbon/graphene composite as high-performance anode for potassium storage. Electrochim. Acta **377**, 138093 (2021). https://doi.org/10.1016/j.electacta.2021.138093

[7] M. Chen, W. Wang, X. Liang, S. Gong, J. Liu et al., Sulfur/oxygen codoped porous hard carbon microspheres for high-performance potassium-ion batteries. Adv. Energy Mater. **8**(19), 1800171 (2018). https://doi.org/10.1002/aenm.201800171

[8] Z. Qiu, K. Zhao, J. Liu, S. Xia, Nitrogen-doped mesoporous carbon as an anode material for high performance potassium-ion batteries. Electrochim. Acta **340**, 135947 (2020). https://doi.org/10.1016/j.electacta.2020.135947

[9] J. Chen, Y. Cheng, Q. Zhang, C. Luo, H.Y. Li et al., Designing and understanding the superior potassium storage performance of nitrogen/phosphorus co-doped hollow porous bowl-like carbon anodes. Adv. Funct. Mater. **31**(1), 2007158 (2020). https://doi.org/10.1002/adfm.202007158

[10] L. Tao, Y. Yang, H. Wang, Y. Zheng, H. Hao et al., Sulfur-nitrogen rich carbon as stable high capacity potassium ion battery anode: performance and storage mechanisms. Energy Storage Mater. **27**, 212-225 (2020). https://doi.org/10.1016/j.ensm.2020.02.004

[11] X. Qi, K. Huang, X. Wu, W. Zhao, H. Wang et al., Novel fabrication of N-doped hierarchically porous carbon with exceptional potassium storage properties. Carbon **131**, 79-85 (2018). https://doi.org/10.1016/j.carbon.2018.01.094

[12] D. Li, X. Ren, Q. Ai, Q. Sun, L. Zhu et al., Facile fabrication of nitrogen-doped porous carbon as superior anode material for potassium-ion batteries. Adv. Energy Mater. **8**(34), 1802386 (2018). https://doi.org/10.1002/aenm.201802386

[13] G. Xia, C. Wang, P. Jiang, J. Lu, J. Diao et al., Nitrogen/oxygen co-doped mesoporous carbon octahedrons for high-performance potassium-ion batteries. J. Mater. Chem. A **7**(19), 12317-12324 (2019). https://doi.org/10.1039/c8ta12504j

[14] X. Lu, X. Pan, Z. Fang, D. Zhang, S. Xu et al., High-performance potassium-ion batteries with robust stability based on N/S-codoped hollow carbon nanocubes. ACS Appl. Mater. Interfaces **13**(35), 41619-41627 (2021). https://doi.org/10.1021/acsami.1c10655

[15] H. Ding, J. Zhou, A.M. Rao, B. Lu, Cell-like-carbon-micro-spheres for robust potassium anode. Natl. Sci. Rev. **8**(9), nwaa276 (2021). https://doi.org/10.1093/nsr/nwaa276

[16] K. Lei, C. Wang, L. Liu, Y. Luo, C. Mu et al., A porous network of bismuth used as the anode material for high-energy-density potassium-ion batteries. Angew. Chem. Int. Ed. **57**(17), 4687-4691 (2018). https://doi.org/10.1002/anie.201801389

[17] F. Ming, H. Liang, W. Zhang, J. Ming, Y. Lei et al., Porous MXenes enable high performance potassium ion capacitors. Nano Energy **62**, 853-860 (2019). https://doi.org/10.1016/j.nanoen.2019.06.013

[18] C. Zhang, Y. Xu, M. Zhou, L. Liang, H. Dong et al., Potassium prussian blue nanoparticles: a low-cost cathode material for potassium-ion batteries. Adv. Funct. Mater. **27**(4), 1604307 (2017). https://doi.org/10.1002/adfm.201604307

[19] R. Zhang, J. Bao, Y. Pan, C.F. Sun, Highly reversible potassium-ion intercalation in tungsten disulfide. Chem. Sci. **10**(9), 2604-2612 (2019). https://doi.org/10.1039/c8sc04350g

[20] C. Lv, W. Xu, H. Liu, L. Zhang, S. Chen et al., 3D sulfur and nitrogen codoped carbon nanofiber aerogels with optimized electronic structure and enlarged interlayer spacing boost potassium-ion storage. Small **15**(23), 1900816 (2019). https://doi.org/10.1002/smll.201900816

[21] Y. Xu, C. Zhang, M. Zhou, Q. Fu, C. Zhao et al., Highly nitrogen doped carbon nanofibers with superior rate capability and cyclability for potassium ion batteries. Nat. Commun. **9**, 1720 (2018). https://doi.org/10.1038/s41467-018-04190-z

[22] W. Qiu, H. Xiao, Y. Li, X. Lu, Y. Tong, Nitrogen and phosphorus codoped vertical graphene/carbon cloth as a binder-free anode for flexible advanced potassium ion full batteries. Small **15**(23), 1901285 (2019). https://doi.org/10.1002/smll.201901285

[23] K. Han, F. An, Q. Wan, L. Xing, L. Wang et al., Confining pyrrhotite Fe_7S_8 in carbon nanotubes covalently bonded onto 3D few-layer graphene boosts potassium-ion storage and full-cell applications. Small **17**(12), 2006719 (2021). https://doi.org/10.1002/smll.202006719
